# Supplementary material for: Are carbon emissions trading and green financial instruments synergistic? -Comprehensive quantitative research based on content analysis
Source: PLoS One. 2024 Mar 7;19(3):e0298601. doi: 10.1371/journal.pone.0298601 (PMC10919601; doi:10.1371/journal.pone.0298601)
Supplement: S1 Data — (ZIP) [file pone.0298601.s001.zip › Data/地级市Z_F工作报告.zip!/╦─┤¿/─╧│Σ╩╨/└┤╫╘╣1⁄2╓┌║┼í╛┬φ┐╦╩2╛▌═°í┐_2017.docx]

各位代表：

　　我代表市人民政府向大会报告工作，请予审议，并请市政协各位委员和其他列席会议的同志提出意见。

一、过去五年工作回顾

　　市五届人大一次会议以来的五年，是南充发展史上极为特殊、极其复杂、极不平凡的五年。面对多重困难叠加、多重任务交织的严峻考验，我们始终保持定力抓发展、攻坚克难求突破、负重前行促跨越，全市经济社会持续健康发展。地区生产总值在全省地级市中率先跨入“千亿俱乐部”、突破1600亿元；全社会固定资产投资、一般公共预算收入实现翻番；规上工业增加值年均增长11%，社会消费品零售总额年均增长13.8%，城镇和农村居民人均可支配收入年均分别增长11%、12.1%；9县（市、区）地区生产总值均突破百亿元大关；全市城镇化率年均增长1.5个百分点、达到45%，南充改革发展和现代化建设事业取得新成就。

　　过去五年， 我们强化产业培育，实体经济不断壮大。 工业向集约化迈进，总投资70亿元的吉利新能源商用车项目落户建设，首期投资30亿元的鑫达新材料项目建成投产，工业园区建成区面积突破100平方公里、工业集中度达到78%。农业向精细化发展，我市成为全省唯一的全国农业改革与建设试点示范区，有机农产品基地规模居西部第一，粮食总产量连续14年居全省首位。服务业向新业态衍变，现代金融、现代物流、电子商务快速发展，南充现代物流园建成开园，我市成功申报为全国首批信息消费试点城市、区域流通节点城市，阆中古城、朱德故里景区成功创建为国家5A级旅游景区。

　　过去五年， 我们强化基础夯实，城乡面貌不断改善。立体交通网络基本形成，兰渝铁路南充段建成通车，建成高速公路增至10条、里程居全省第二，高坪机场开通航线增至12条，“一港三码头”开港营运。水利基础设施加快建设，解元水库、油房沟水库、九龙潭水库全面完工。通信基础设施日益完善，“光网南充”全面建成。城镇建设步伐加快，中心城区建成区面积超过120平方公里、常住人口超过120万人，成为全省首批3个Ⅱ型大城市之一；6个县城、23个国省重点小城镇建设扎实推进，1172个幸福美丽新村基本建成。数字城管系统投入使用。成功创建国家生态文明先行示范区、国家园林城市，全市森林覆盖率达到39.96%。

　　过去五年， 我们强化改革开放，发展动能不断集聚。 重点改革有序推进，农村“七权”同确基本完成，9县（市、区）农村产权交易中心挂牌成立；成功申报公立医院改革国家联系试点城市，公立医院药品加成全面取消；东风南充汽车公司顺利转制；南充农商行挂牌开业；投融资体制、新型城镇化和供给侧结构性改革等扎实推进。创新创业活力得到激发，西华师范大学科技园成功创建川东北首家省级大学科技园，南充创业小镇影响力持续扩大，全市民营经济市场主体突破20万户。开放合作成效明显，成功举办中国四川国际文化旅游节、中国南充国际木偶艺术周、C21论坛等活动，西博会、融渝投资促进等活动成效明显，全市招商引资到位资金累计超过2300亿元。

　　过去五年， 我们强化社会建设，民生福祉不断提升。公共财政在民生领域投入超过1370亿元，向社会承诺的民生工程、民生大事全面完成。脱贫攻坚首战全胜，南部、蓬安脱贫摘帽顺利通过省级评估验收，全市326个贫困村完成退出、67.3万贫困人口实现脱贫。基本公共服务均衡发展，新建、改（扩）建学校（幼儿园）767所；西南石油大学（南充）恢复本科招生；川北医学院附属医院新院区、市中心医院门诊住院大楼、市中医院综合楼和240所乡镇卫生院、1446个村卫生室建成投入使用；成功创建国家公共文化服务体系示范区，实现“4+ 2”公共文化服务网络全覆盖。社会保障体系不断完善，新增城镇就业保持稳定态势；城乡居民医疗保险制度全面整合，医疗、失业、工伤、生育、企业职工养老保险实现市级统筹，社会救助体系更加健全；建成保障性住房和棚户区改造安置住房20.5万套；新（改）建农村公路8887公里，解决了201.4万农村人口和31.4万学校师生饮水安全问题。社会治理持续加强，“法治南充”“平安南充”建设深入开展，安全生产和食品药品安全形势持续好转，社会大局保持和谐稳定。

　　过去五年， 我们强化职能转变，自身建设不断加强。深入开展党的群众路线教育实践活动、“三严三实”专题教育和“两学一做”学习教育，着力建设人民满意政府。自觉接受市人大及其常委会的法律监督、工作监督和市政协的民主监督，组建市委市政府法律顾问团，建立“一对一”法律顾问制度，切实将政府工作纳入法治化轨道。全面推行政府部门目标绩效管理，持续开展社会满意度测评，挂图作战推动重大决策部署落地落实。大力推进简政放权，行政审批在法定时限基础上提速65%。全面深化政务公开，在全国率先探索政务公开标准化体系建设。持续用力正风肃纪，“四风”问题得到有效遏制；深刻汲取南充拉票贿选案惨痛教训，猛药去疴，扶正祛邪，政治生态和社会风气明显好转。

　　与此同时，国防动员、人民防空和双拥优抚工作扎实推进，行政监察、审计监督、国资监管、检验检疫、统计调查、计划生育、国土、工商、质监、海关、气象、档案、地方志、保密、国安、信访、邮政、贸促、外事、侨务、港澳台等工作取得新成绩，民族宗教、精神文明、慈善、老龄、妇女儿童、青少年、残疾人、工会、红十字、爱国卫生等工作发挥了积极作用。

　　各位代表！惟其艰难，更显勇毅；惟其磨砺，始得玉成。成绩来之不易，经验弥足珍贵。实践启示我们：

　　———必须坚定不移地把推动发展作为第一要务。我们坚持以经济建设为中心，主动适应经济发展新常态，保持专注发展定力，全力以赴稳增长调结构促转型，推动经济发展行稳致远、稳中向好。实践证明，只有持续抓好科学发展、加快发展，才能战胜各种艰难险阻，才能做到量增质更优，才能推动南充后发赶超。

　　———必须坚定不移地把项目攻坚作为主要抓手。我们坚持以项目统揽全局工作、组织经济工作、推动政府工作，挂帅出征抓项目，挂图作战建项目，挂责问效推项目，形成了拉动经济社会发展的强大引擎。实践证明，只有持续抓好项目牵引、项目推动，才能促进工作落地落实，才能汇聚众力激发活力，才能进中突破强势发展。

　　———必须坚定不移地把改革开放作为动力源泉。我们坚持以深化改革突破体制束缚，以扩大开放厚植发展优势，努力讲好南充故事，积极传播南充声音，主动展示南充形象。实践证明，只有持续抓好深化改革、开放合作，才能聚集资源富集资本，才能挖掘潜力增添动力，才能高点起步跨越转型。

　　———必须坚定不移地把改善民生作为最大责任。我们坚持以服务民生为根、以推动共享为本，解决了一大批涉及群众切身利益的突出问题，让发展成果更多更好地惠及全市人民。实践证明，只有持续抓好民生保障、民生改善，才能维护社会公平正义，才能促进社会安定和谐，才能保证发展不离初衷。

　　———必须坚定不移地把厉行法治作为根本准则。我们坚持将政府工作置于市委的坚强领导下，把廉洁从政作为政府工作的钢要求、铁纪律，自觉运用法治思维和法治方式推进工作，切实做到法定职责必须为、法无授权不可为。实践证明，只有持续抓好依法治市、健全法治，才能不断增强政府公信力，才能更好凝心聚力谋发展，才能树立良好政务形象。

　　各位代表！五年风雨兼程、攻坚克难，五年冬耕夏耘、春华秋实。五年来取得的成绩，是省委省政府和市委坚强领导的结果，是市人大市政协监督支持的结果，饱含着全市干部群众的辛劳与付出，凝聚着社会各界人士的智慧和力量。在此，我代表市人民政府，向全市人民，向大力支持政府工作的各位代表、各位委员，向各民主党派、工商联、无党派人士和人民团体，向驻市解放军、武警官兵和政法干警，向所有关心和支持南充发展的海内外朋友们表示衷心的感谢，并致以崇高的敬意！

　　各位代表！我们也清醒地认识到，南充发展还面临不少困难和问题：人口多资源少、底子薄基础差、财力弱负担重的市情没有根本改变；经济结构不优、创新能力不足、发展质量不高的问题未能完全解决；初级阶段特征明显、二元结构矛盾突出、民生领域欠账较多的现状仍然客观存在；思想观念不新、发展理念不适应、闭守心态突出等障碍尚未彻底消除；转变工作作风、优化政务环境、加强廉政建设任重道远。这些问题不容回避，我们将本着对党和人民高度负责的态度，以更大的决心、更大的力度，采取有效措施，切实加以解决。

二、未来五年的奋斗目标和主要任务

　　各位代表！大城崛起，未来已来！市第六次党代会确立了引领南充新发展、开创南充新未来的“155发展战略”，这是置身新的历史方位顺势而为的先机抉择，这是站在新的历史起点乘势而上的科学考量，这是承载新的历史使命逆势而进的勇毅担当，吹响了南充大城崛起的冲锋号角，擂响了南充奋力作为的进军战鼓，开启了南充强势突破的宏伟征程。“南充新未来·成渝第二城”的战略目标，集中反映了全市人民的共同愿望，高度浓缩了川北重镇的精神气质，清晰描绘了未来南充的壮美图景，必将激发人心思上、人心思进的昂扬斗志，必将汇集万众一心、团结一致的磅礴气势，必将凝聚无坚不摧、无往不胜的强大力量。做大做强“五大千亿产业集群”，是遵循产业演进规律、提升城市实力的战略审视，必将推动南充产业飞跃发展、加速排浪式升级。突破突围“五大板块重大工程项目”，是优化投资结构、引爆南充发展的战略选择，必将推动南充蓄力高位起跳、加速跨越式赶超。

　　承载新的历史使命，肩负新的历史重任，我们必须汇聚全市之力，拿出冲天干劲，以非常之为成非凡之事，以超常之举建超常之功，努力实现五年初见成效、十年大见成效，全力重塑区域经济格局、重振川北重镇雄风！

　　今后五年， 我们的指导思想是：高举中国特色社会主义伟大旗帜，深入学习贯彻以习近平同志为核心的党中央治国理政新理念新思想新战略，紧紧围绕“五位一体”总体布局和“四个全面”战略布局，全面贯彻落实省委治蜀兴川系列战略部署和市第六次党代会精神，大力实施“155发展战略”，强化责任担当，奋力攻坚克难，推动南充在成渝之间强势崛起、次级突破，在成渝之后率先跨越、一马当先，为同步全面建成小康社会、加快建设成渝第二城而努力奋斗！

　　今后五年， 我们的主要预期目标是：主要指标增速高于全国、快于全省，在全面实现市第六次党代会确定的“一个全面、两个突破、三个翻番”基础上，力争到2021年，地区生产总值达到2250亿元，全社会固定资产投资达到3100亿元，一般公共预算收入达到190亿元，规上工业企业销售收入达到4600亿元，社会消费品零售总额达到1350亿元，城镇和农村居民人均可支配收入分别达到38100元、17800元；经济质量和效益明显提升，产业结构更加优化，城乡面貌、人民生活、生态环境持续改善，成渝第二城魅力彰显。

　　今后五年， 我们的主要任务是：紧紧围绕市第六次党代会确立的“南充新未来·成渝第二城”五大奋斗方向，聚焦聚力“五大千亿产业集群”，加速加力“五大板块重大工程项目”，全面推进基础设施“十大建设”、产业发展“十大项目”、民生改善“十大工程”，按照项目化、工程化、具体化的要求，铺排工作、调度资源、推进落实，主要着力点包括：

　　（一）加快打造经济强市，着力提升区域竞争力。经济实力是成渝第二城的核心支撑。我们将坚持“以产立城、以业兴市”，夯实发展支撑，努力建设产业兴盛、实力强劲的城市。持续壮大汽车汽配产业集群。坚持把吉利新能源商用车项目作为促进实体经济发展的“一号工程”，推动尽早投产达产，实现年产整车10万台以上、产值超200亿元。围绕南充新能源汽车产业园建设，大力招引一批新能源汽车电机、电控等核心部件和变速箱、车桥总成等配套企业，培育产值超3亿元的机械汽配小巨人企业110户以上。建成吉利南充新能源商用车研究院、国家级企业技术中心和国家新能源汽车检验检测中心，打造西部新能源商用车研发生产基地。力争全市汽车汽配产业集群产值超过1100亿元。持续壮大油气化工产业集群。推动晟达、石达、联成等项目投产见效，着力建设高分子材料产业园、化纤产业园和鑫达新材料二期项目，沿链引进纳米新材料、清洁能源装备制造等行业领军企业，推动南充经开区加速转型、高端发展，全力打造西部石化基地、新材料产业基地和国家清洁能源发展基地。加快推进中国西部生物医药产业园、西充太极生物医药谷等项目建设，推动生物质能、生物医药等战略性新兴产业发展壮大，建成西部生物医药基地。力争全市油气化工产业集群产值超过800亿元。持续壮大丝纺服装产业集群。做大高坪都京丝纺服装产业园、嘉陵丝纺服装产业园等重点园区，打造西部最大的丝纺服装研发生产基地和产品集散中心。联动发展栽桑养蚕、丝绸文化、丝绸旅游等产业，建成西部最大的蚕桑综合开发利用基地、中国蚕桑丝绸文化旅游名城。深挖“百年六合”“依格尔丝绸”等品牌价值，打响“中国绸都”“丝路源点”品牌，推动“南充造”丝纺服装产品拓展国际市场。力争全市丝纺服装产业集群产值超过660亿元。持续壮大现代物流产业集群。丰富完善南充现代物流园业态，新引进有实力的领军型物流企业3家以上，建成成渝城市群重要的进出口物流基地。以高坪机场、嘉陵江航道和市域多条高速公路、铁路为依托，加强商贸流通、供销、邮政等物流服务设施建设，形成公路、铁路、水运、航空多式联运的立体式物流网络。有序推进各县（市、区）配送型物流中心建设，加快完善城乡物流配送体系。积极推进保税物流中心、海关特殊监管场站建设，创建跨境电商平台和进口指定口岸，争创南充综合保税区，建成现代物流枢纽城市和区域物流中心城市。力争全市现代物流产业集群产值超过700亿元。持续壮大现代农业产业集群。高起点编制现代农业发展区域规划，合理布局各县（市、区）现代农业发展主攻方向。加强与中国农大等高校的深度合作，构建新型农业科技研发联盟，培育招引一批国内一流的农业高科技龙头企业。加快国家农业科技园和中法农业科技园建设步伐，推动国家现代农业示范区扩面提档。深化农业供给侧结构性改革，实现农业产业链延伸、价值链提升。建设全国有机农产品价格指数平台和交易中心，打造一批特色农产品区域公共品牌，建成有机农业强市。力争全市现代农业产业集群产值超过1100亿元。

　　同时，扎实推进南充云计算中心和三环电子四期、五期等项目建设，实施“互联网+”行动计划，积极发展集成电路、信息网络安全、信息软件服务等电子信息产业，以跨界融合催生新业态，全面拓展信息经济空间；以燕京啤酒、张飞牛肉、保宁醋为龙头，推进轻工食品产业提档优化、升级发展；加快全域旅游资源开发，创建特色旅游品牌，打造国内一流国际知名的旅游目的地。

　　（二）加快打造中心城市，着力提升城市影响力。中心城市是成渝第二城的首要标志。我们将坚持城市形态、城市业态、城市文态“三态合一”，努力建设大气精致、魅力独具的城市。有序拓展城市空间。统筹谋划跨江东进、拥江发展、三城同构，优化市域空间总体布局，高水平推进下中坝、清泉坝等城市新区建设，做大现有中心城区规模；全力推进西充、蓬安撤县设区，按照区域同规、产业同链、基础同网、公共服务同享的基本方略，加快“南西蓬”一体化发展，着力形成“一城五区”城市发展格局，力争中心城区建成区面积超过200平方公里、常住人口超过200万人，基本奠定Ⅰ型大城市框架。积极完善城市功能。大力实施城市畅通工程，建成都京港嘉陵江大桥和将军路嘉陵江大桥；大力实施生态绿化工程，打造西山城市公园和人民广场休闲公园，推进蓬安漫滩湿地公园和西充海绵城市建设；大力实施功能配套工程，建成“印象嘉陵江”城市会客厅、国际会展中心和高铁商务区、“顺庆府”特色商业街区，加快建设南充国际高中、区域职业教育城、市中心医院江东医院；提高市政设施现代化水平，构建清洁高效的现代能源体系、安全优质的供水保障体系，加快地下综合管廊建设，推动实施老旧住宅小区电梯改造，合理规划建设中心城区停车场（库），提高城市承载力、宜居度、美誉度和市民满意度。不断优化城市业态。持续深化城市有机更新，提升中心城区集聚功能和辐射带动能力，加快推进中心城区传统商贸退批进零、工业企业退城入园、产业业态退二进三，推动城市形态和城市业态同步优化提升。大力发展楼宇经济、总部经济，积极引进现代金融、旅游服务、商贸会展、文化创意等业态，建成区域商贸中心、金融中心。推进智慧政务云为主体的基础平台应用，加快智慧城市建设。提高乡村规划建设水平，建设一批高品质小城市、中心镇和美丽乡村。

　　（三）加快打造交通枢纽，着力提升交通支撑力。交通枢纽是成渝第二城的重要基础。我们将坚持水、陆、空“三线并举”，打造畅达区域、通向全国、连接世界的国家次级综合交通枢纽，努力建设通达便捷、互联高效的城市。提升航运综合能力。扎实推进嘉陵江高等级航道建设，加快嘉陵江渠化步伐，提升航道全天候通航能力；大力推进港口建设，建成阆中、南部、蓬安旅游客运码头，打通港区进出运输通道，形成结构优化、便捷互通的现代港口体系，建成区域重要内河港口城市；加强嘉陵江智慧航运建设，推动港口与港口综合配套服务区、物流园区有效衔接，实现港区、城区、园区三区互动发展。完善陆上交通网络。加快铁路通道建设，启动建设蓉京高铁、汉巴南快铁，谋划建设连通西蓬、覆盖三区的城市轨道交通和南渝城际铁路；继续大力实施“南充交通三年推进计划”，建成南绵、营达、南充二绕等高速公路，加快建设南潼、阆仪营、成南（扩容）等高速公路，打好国省干线改造、快速通道建设、农村公路联网、渡改桥“四大战役”，建成顺蓬营、顺仪、西顺嘉一级公路，完善城市交通网、慢行交通系统和中心城区对外通道，形成“南西蓬一体、市域半小时、成渝一小时”交通圈。打造区域空中走廊。建成高坪机场航站楼，科学论证推动阆中机场建设；加大与相关航空公司合作力度，盘活用好时刻与航线资源，多措并举拓展吸纳周边市县客源，提升机场服务水平，不断提高旅客和货物吞吐量；加强与重要节点城市航线直通，争取开通国际航线，力争将高坪机场建成川东北地区重要的航空门户。

　　（四）加快打造开放高地，着力提升要素集聚力。改革开放是成渝第二城的必由之路。我们将坚持提升双向开放水平，深入推进改革攻坚，在更宽领域、更高层次、更大范围集聚要素资源，努力建设要素涌流、活力迸发的城市。持续深化重点改革。坚持以供给侧结构性改革为主线，以经济体制改革为牵引，围绕“三权分置”深化农业农村改革，围绕提高竞争力和资源配置效率深化国资国企改革，围绕简政放权、放管结合、优化服务深化行政管理体制改革，围绕激发投资活力、畅通融资渠道深化投融资体制改革，统筹抓好社会治理、社会保障等基础性改革，扎实推进公立医院改革、中小城市综合改革等国省改革试点，释放更多改革红利。大力实施创新驱动。加强源头创新、开放创新、协同创新，激发全社会的创新活力和创造潜能，打造川东北创客中心。创建国家高新技术产业开发区，打造一批产学研用紧密结合的协同创新平台，建成一批国省级孵化器。加速创新成果转化，高标准建设科技成果转化服务平台，定期举办科技成果对接交易活动，以科技成果转化促进实体经济发展。深入实施“嘉陵江英才工程”，引进培育综合管理人才、行业领军人才和实用技术人才1000名以上，努力建设人才强市。积极推进开放合作。以打造内陆型改革开放新高地为目标，深度融入“一带一路”、长江经济带和成渝发展，促进要素有序流动、资源高效配置、市场深度融合，建成成渝城市群开放“桥头堡”。推进外贸发展转型升级，深入实施外贸主体培育、出口基地创建、出口品牌建设“三大工程”，力争全市外贸进出口总额累计超过18亿美元。提升招商引资质量，加强产业招商、以商招商，引进一批大项目、好项目落户南充，力争全市招商引资到位资金累计超过3000亿元。

　　（五）加快打造生态屏障，着力提升环境承载力。生态良好是成渝第二城的鲜明特质。我们将坚持生态优先、生态打底、生态强市，努力建设生态友好、绿色低碳的城市。推进“嘉陵江生态屏障”建设。全域启动嘉陵江（南充段）综合保护开发，实行最严格的生态保护制度，科学划定生态保护红线，加强水环境监测和预警体系建设。持续抓好江河治理，不断强化江河源头和水源涵养区生态保护。加大嘉陵江岸线原貌保护力度，加快推进沿江自然保护区、湿地公园、滨江区域等重要节点建设，着力打造嘉陵江绿色生态走廊。积极创建国家内河流域综合保护开发示范区。强化城乡生态建设保护。守住“山上、天上、水里、地里”生态底线，把好山好水好风光融入城市。严格环境保护执法，持续开展大气污染防治，加大城市烟尘、建筑扬尘、机动车尾气等防治力度，严控秸秆焚烧；持续开展水污染防治，加强饮用水水源地和良好水体保护，加大城镇生活污水处置力度，大力整治农村面源污染和地下水污染；持续开展土壤污染防治，全面开展城镇生活垃圾分类处理和无害化处理，实现土壤分类利用。大力实施“大规模绿化南充行动”，加快推进生态修复、植树造林、退耕还林、绿色通道、城乡一体绿化等工程建设，努力实现城市、集镇、农村“三位一体”，水网、路网、林网“三网合一”，生态、产业、景观“三林共建”，整体推进全市绿化。全面落实保护森林资源任期目标责任制，力争森林覆盖率达到并稳定在41%以上。完善城市生态文明体系。大力推进绿色、低碳、循环发展，建立集约节约使用资源的激励和约束机制，实行最严格的耕地保护制度。新建一批循环经济示范基地和低碳工业园区，培育一批生态文明示范单位，全市产业低碳化、交通清洁化、建筑节能化、主要污染物减量化、可再生能源利用规模化水平明显提升。倡导和普及绿色生活方式。建立生态文明绩效评价制度，严格执行生态环境损害责任追究制度。建成国家生态市。

　　（六）加快打造幸福家园，着力提升发展凝聚力。人民幸福是成渝第二城的发展归宿。我们将坚持以人民为中心，深入推进生活品质共建共享，努力建设幸福美好、平安和谐的城市。坚决打赢脱贫攻坚硬仗。按照“四年攻坚、一年巩固、一年提升”和“不落下一户一人”的要求，紧紧围绕“两不愁、三保障”“四个好”目标，深入实施“五大扶贫工程”，全面落实“五个一批”行动计划，继续坚持“五个一”帮扶机制，确保如期实现现行标准下贫困县全部摘帽、贫困村全部退出、贫困户全部脱贫，2020年与全国全省同步全面建成小康社会。全面提升民生保障水平。逐年解决一批民生难题，办好一批民生大事，努力让人民群众生活更有获得感、更有幸福感。大力实施就业创业促进工程，完善就业创业培训体系，新增城镇就业33万人。多渠道增加城乡居民收入，着力提高低收入群体的收入水平。有序推进基本公共服务均等化，推动教育有质量全面发展，基本普及学前三年教育，实现县域内义务教育基本均衡发展，普及高中教育，支持发展高等教育，做优做强职业教育，加强特殊教育，大力发展民办教育；促进医疗卫生扩量提质，不断提升城乡医疗卫生服务水平。加强社会保障，扩大各类社会保险覆盖面，构建覆盖城乡全体居民、更加公平可持续的社会保障体系。切实增强社会治理能力。深入推进依法治市，自觉运用法治思维和法治方式推进工作，纵深推进法治政府、法治社会、法治城市建设。加强流动人口管理服务，引导支持社会组织参与社会治理。深化“平安南充”建设，完善立体化社会治安防控体系，深入推进依法治网，切实抓好安全生产，保障食品药品安全，打造最具安全感城市。

　　各位代表！百舸争流千帆竞，只争朝夕莫等闲。今后五年，将是南充厚积薄发、强势崛起的五年，将是南充乘风破浪、阔步前行的五年。只要我们以“开局就是决战、起步就是冲刺”的精神状态，以“功成不必在我、功成必定有我”的使命担当，苦干实干、求新求变，久久为功、善作善成，就一定能够绘就南充新未来的美丽画卷，就一定能够谱写成渝第二城的辉煌篇章！

三、2017年工作安排

　　2017年是新一届市政府工作的开局之年，也是全面实施“155发展战略”、加快建设“南充新未来·成渝第二城”的发力之年。今年政府工作的总体要求是：全面贯彻落实中央、省委和市委决策部署，始终保持专注发展、转型发展的战略定力，牢固树立和贯彻落实新发展理念，主动适应把握引领经济发展新常态，按照市委“坚决保持‘三个高于’的稳中求进总基调、坚决实现‘三大突破’的跳起摸高总目标、坚决打赢‘三场必胜’的攻坚克难总体战”的工作思路，统筹做好稳增长、促改革、调结构、惠民生、防风险各项工作，为开创南充新未来、建设成渝第二城奠定坚实基础。

　　全年经济社会发展主要预期目标是：地区生产总值增长7.6%，全社会固定资产投资增长12%，一般公共预算收入增长7%，规上工业增加值增长9.5%，社会消费品零售总额增长12%，服务业增加值增长8.6%，城镇和农村居民人均可支配收入分别增长8.5%、10%，城镇化率提高1.2个百分点。

　　围绕上述目标，今年将重点抓好八个方面工作：

　　（一）坚持产业兴市、工业强市，加快推动产业集群发展

　　加快发展汽车汽配产业。 大力实施“千亿百家计划”和“四个一工程”，启动建设天喜新能源车用空调研发生产基地，加快西南汽车模具、中国重汽南充基地等项目建设；推动吉利新能源商用车项目竣工投产，形成年产新能源商用车10万台的产能；延展汽车零部件配套产业链条，新增产值超3亿元的机械汽配小巨人企业5家以上。出台新能源汽车推广应用方案，在市内公交车、出租车等行业推广使用一批“南充造”新能源汽车，建成新能源汽车充（换）电设施37处以上，开通新能源公交专线2条以上。

　　加快发展油气化工产业。开工建设恒阳伟业、青龙化工等项目，加快晟达、石达、联成等项目实施进度，大力推进宏泰生化、飞龙化工、顺城盐化搬迁改造，确保精对苯二甲酸等项目投产见效，中石化液化天然气（LNG）阆中基地项目全面竣工，力推昂鼎科技、重庆医药化工等项目签约落地。加快建设中国西部生物医药产学研基地、中国西部生物医药产业园，开工建设西充太极生物医药谷等项目。推动鑫达新材料一期全面达产、二期开工建设。

　　加快发展丝纺服装产业。扎实推进嘉美印染纺织生产线等项目建设，支持六合、依格尔、富安娜、金富春等龙头企业技改扩能、拓展市场，鼓励企业组建丝纺服装产业技术创新联盟。加大丝纺服装品牌创建力度，积极申报“中国名牌”“中国驰名商标”“高档丝绸标志”和地理标志产品。推动“中国网库”丝绸产业单品电商平台建设，确保年内投入营运。启动建设都京丝绸特色小镇、丝绸博物馆等项目，加快建设都京丝绸文化产业园，着力打造丝绸文化旅游精品。

　　加快发展现代物流产业。启动建设南充现代物流园城际物流配送中心、中国物流南充分中心、南充跨境电商平台和进口产品国家体验馆等项目，加快建设川东粮油储备调控中心，确保川东北粮食物流中心等项目全面竣工，南充保税仓、海关监管场站投入使用。加快推进顺庆城市共同配送试点、西充城乡双向流通体系建设。推动浙江传化、南鑫国际、友信崧锋等企业发展壮大，继续引进知名物流企业，支持本地企业争创国家A级物流品牌。全社会物流总额超过3400亿元、物流业增加值突破100亿元。

　　加快发展现代农业。启动实施“三百示范工程”，高标准建设千公里现代循环农业示范带，建成现代循环农业产业园区40个、现代农业新产业新业态示范基地30个，培育产值过亿元龙头企业12家、过10亿元龙头企业3家。着手筹建亚洲有机农业研发中心，新认证有机农产品基地5万亩。加速推进亭子口灌区工程前期工作，扎实推进升钟灌区二期、营山金鸡沟水库建设；新增高标准农田25万亩、有效灌面30万亩，提高农业综合生产能力。

　　（二）坚持项目推动、投资拉动，突出抓好重点板块项目

　　突出抓好重大城建项目。 认真开展将军路嘉陵江大桥、滨江北路互通等项目前期工作，开工建设“印象嘉陵江”城市会客厅、高铁商务区等重点项目，加快推进都京港嘉陵江大桥、滨江中路互通、顺庆地下综合管廊、西充海绵城市等项目建设，全面完成白塔嘉陵江大桥维护、金融大道、北部新城一路等项目建设。启动江东大道、玉带路改造提升，推进人民广场休闲公园、西山城市公园等重要节点改造提升，实施中心城区沿江两岸、机场和高速公路出入口、火车站站前广场等区域绿化、美化、亮化，着力打造“顺庆府”、白马水街等特色街区。启动城市总体规划修编论证，编制完成“南西蓬”一体化发展规划、嘉陵江流域空间规划，完成蓬安撤县设区申报工作。

　　突出抓好重大交通项目。 认真做好蓉京高铁、汉巴南快铁项目前期工作。加快建设南绵、营达高速公路，开工建设南潼、南充二绕和成南（扩容）等高速公路。加快推进定升、顺蓬营等干线公路建设，力争顺仪、西顺嘉等干线公路年内开工。加快建设高坪机场航站楼，确保主体结构工程完工；新开辟航线3条。扎实推进嘉陵江航运配套工程建设，完善都京港作业区一期工程、经济开发区专用码头配套设施。

　　突出抓好重大民生项目。 启动建设南充国际高中、市体育中心改造、南充职业技术学院新校区二期工程等项目，新建、改（扩）建中小学、幼儿园校舍40万平方米，建成标准中心校18所、农村义务教育寄宿制学校42所。力争启动建设市川剧艺术中心、美术馆，加快建设市博物馆、市档案馆，全面建成“四馆一中心”。启动建设川北医学院附属嘉陵医院、市中医院新院区和川东北康养中心，加快实施市中心医院江东医院、川北医学院附属医院新院区住院大楼等项目，确保市儿童医院、市中心血站检验大楼投入使用；实施县级医疗卫生机构项目11个。加快推进营山饮用水工程、市第四自来水厂建设，启动实施顺庆燕儿窝旧城改造等项目，完成城镇危旧房棚户区改造13482套。

　　突出抓好旅游三产项目。 启动实施嘉陵江综合保护生态休闲旅游带、蓬安相如湖国家级旅游度假区等重点项目，推动阆中创建全国休闲旅游示范城市、南部升钟湖创建国家级旅游度假区、朱德故里景区建设全国红色旅游示范区，力争创建4A级旅游景区1个、3A级旅游景区5个。大力实施服务业“三百工程”，启动建设国际会展中心、南充国际商贸城等项目，统筹推进南门坝商圈、西华商圈提档升级，加速繁荣江东商圈、南湖商圈。深入实施“触网触电”工程，扎实推进西充、仪陇全国电子商务进农村综合示范建设，加速推进顺庆电商进社区示范推广等项目建设。力争启动建设“一库两中心”，支持金融机构设立区域总部，积极打造现代金融集聚区。

　　突出抓好产业园区项目。 加快建设以南充新能源汽车产业园、南充经开区为龙头的10个工业园区，力争全市工业园区新增面积5平方公里。扎实推进国家现代农业示范区、国家农业科技园、中法农业科技园3个“国字号”园区建设。加快完善南充现代物流园功能配套，确保保税中心连接线、铁路专用线建成竣工。加大国省重点园区创建力度，新增省级重点（试点）工业园区2个。着力打造都京丝纺服装产业园等一批专业园区，确保新增年销售收入超300亿元产业园区2个、超500亿元1个。

　　（三）坚持深化改革、扩大开放，充分释放经济内生动能

　　强力推动全面改革。 深入推进供给侧结构性改革，突出抓好房地产去库存，加大住房货币化安置力度，支持进城农民工等刚需群体购房，促进房地产市场健康发展；加大减税降费力度，大力争取富余电量和直购电政策，引导金融机构降低融资成本，努力推动企业降本增效。深化农村产权制度改革，推进农村土地“三权分置”，着力构建新型农业经营体系。深化财税体制改革，进一步完善市区财权、事权相匹配的体制。深化公立医院改革，着眼医药、医疗、医保“三医联动”，完善管理体制和运行机制，鼓励社会资本参与公立医院发展。深化金融体制改革，大力支持农信社改制，完成南部农商行挂牌开业。扎实抓好行政审批、国资国企、生态文明等重点改革，加快推进新型城镇化、中小城市综合改革等国省改革试点，努力创造可复制、可借鉴、可推广的鲜活经验。

　　强力推动招大引强。锁定成渝、长三角、珠三角、环渤海、港澳台等重点区域，瞄准“三类500强”、央企和上市公司，全力引进一批大项目、好项目。积极参加“全球川商返乡发展大会”“中外知名企业四川行”“西部国际投资大会暨进出口展”“渝洽会”等活动，组织开展汽车汽配、油气化工产业等专题投资推介活动，分区域、分产业、分板块开展系列投资促进活动。大力实施“归雁计划”，鼓励有实力、有意愿的乡友企业家回乡投资兴业。全年新签约项目160个以上，实现到位资金512亿元以上。

　　强力推动对外合作。深化与法国、德国等欧洲国家和“一带一路”沿线国家的交流合作，积极拓展友城网络。主动融入四川、重庆自贸区建设，加强与川东北经济区、重庆经济协作区、成渝城市群的区域合作。鼓励企业走出去，支持企业积极参加“万企出国门”活动，引导企业开展境外投资合作、参与国际竞争、拓展海外市场，推动“南充造”优势产品走出去，力争全市外贸进出口总额达到2.3亿美元以上。

　　（四）坚持厚植优势、挖掘潜能，持续激发创新创业活力

　　积极搭建创新平台。 加快建设南充创新驱动发展试验区，启动创建国家高新技术产业开发区，力争川东北军民融合创新产业园落地建设；支持南充经开区、各县（市、区）产业园区建设创业苗圃和科技孵化器，新建创业孵化基地9个。鼓励各类企业与科研院所、高校和创业团队加强资源融通，打造产学研用紧密结合的众创空间。推进西南石油大学（南充）国家大学科技园、西华师范大学省级大学科技园、浙江大学技术转移中心建设，支持川北医学院建成生物医药产业技术研究院、启动创建省级大学科技园，力争设立中国科学院南充产业育成中心、清华大学南充创新中心。

　　积极提升创新能力。 大力实施青年创业促进计划、大学生创业引领计划、农民工返乡创业计划、巾帼创业行动，全年新增青年创业导师100名，开展青年创业培训5000人次。大力实施“嘉陵江英才工程”，引进高层次创新创业人才200名、团队5个。深入实施创新型领军企业培育计划、科技型企业成长计划和企业研发能力提升工程，新增高新技术企业10家。实施重大科技项目30项，重大科技成果转化项目30个。

　　积极营造双创环境。 落实激励科技人员创新创业政策，抓好南充农科院先行先试经验推广。深入推进商事制度改革，加快企业登记“多证合一”改革试点，进一步放宽市场准入，降低创新创业门槛。全年新增各类市场主体1.7万户以上。探索建立科技贷款风险补偿基金，积极引导社会资金支持创新创业，鼓励金融机构组建科技银行。加强知识产权保护，整顿规范市场秩序，构建公平公正的市场环境。广泛开展各类双创大赛，精心办好双创活动周南充活动，持续点燃大众创业、万众创新激情。

　　（五）坚持绿色为底、生态优先，努力夯实绿色生态基底

　　扎实开展环境污染防治。 继续实施“蓝天”行动，全面推进扬尘整治、燃煤控制、秸秆禁烧、废气治理，力争PM2.5年均浓度同比下降2.1%以上。继续实施“碧水”行动，推行“河长制”，确保嘉陵江、西充河、螺溪河、流江河等江河流域和升钟湖等湖库水质持续稳定达标；加强城乡饮用水源地保护，启动城市应急备用水源地建设，确保城市集中式饮用水源地水质100%达标。继续实施“净土”行动，切实加强固体废物、重点工业污染源和农村面源污染治理，让群众呼吸新鲜空气、喝上干净水。

　　扎实推进生态南充行动。 推动嘉陵江综合开发保护立法，完成嘉陵江（南充段）综合保护开发规划编制。大力推进城市立体绿化、邮票绿地、绿道绿廊建设，确保人均公园绿地面积达到12平方米。大力实施天然林保护工程，继续实施荒山造林、封山育林，管护国有林6.5万亩，巩固退耕还林成果83.8万亩。全面落实保护森林资源任期目标责任制，建立森林面积、森林蓄积双增长监测体系，确保全市森林蓄积达到2950万立方米，森林覆盖率达到40%。

　　扎实抓好环保能力建设。 大力推进城镇污水处理基础设施建设，启动顺庆区供水一体化及污水处理、嘉陵工业集中区污水处理一期等项目建设，确保文峰污水处理厂一期、南部污水处理厂二期建成竣工，完成5000吨以上污水处理厂提标改造工程和45个乡镇污水处理设施建设。加大城镇、乡村生活垃圾处理及转运设施建设力度，全面开展城镇生活垃圾分类处理和无害化处理。力争中心城区、县城污水集中处理率分别达到88%、76%，生活垃圾无害化处理率分别达到95%、80%。

　　（六）坚持背水一战、全力冲刺，坚决打赢脱贫攻坚硬仗

　　补齐基础条件短板。 大力实施农村危房改造、易地扶贫搬迁、避灾避险安置“三大工程”，完成1.1万户建档立卡贫困户危房改造、3.48万人易地扶贫搬迁，全力保障贫困群众住房安全。扎实抓好交通、水利、通信等基础设施建设，新（改）建农村公路1200公里，完成渡改桥12座；解决7.8万建档立卡贫困人口饮水安全问题；行政村通宽带比例达到85%；建成扶贫新村300个。

　　推动长效稳定脱贫。新建“脱贫奔康产业园”410个，实现今年拟退出的贫困村全覆盖。积极培育新型农业经营主体，新增农民合作组织200家以上。大力开展贫困家庭就业培训，确保有劳动能力的贫困户至少1人实现就业。加大生态扶贫力度，兑现生态补偿4300万元以上。推动教育、医疗、文化等公共资源向贫困地区倾斜，继续加大对贫困家庭学生资助力度，推行“先诊疗、后结算”就医模式，确保贫困地区广播电视数字化覆盖率超过80%。扎实推进“四好村”创建，力争创建县级以上“四好村”1100个。

　　确保脱贫精准见效。认真落实“六个精准”要求，切实做到因村施策、因户施策、因人施策。加大扶贫资金与其他涉农资金整合力度，全面落实市县两级财政扶贫专项资金预算，确保一般公共预算安排部分在2016年的基础上增长38%以上。深入推行“三个一”调度、“9+5”督查暗访和市级领导蹲点督导机制，确保高坪、嘉陵、仪陇脱贫摘帽，410个贫困村退出，8.3万贫困人口脱贫。

　　（七）坚持富民惠民、共建共享，不断提高人民生活质量

　　切实办好民生实事。 继续加大民生投入，确保财政用于民生的比重超过70%。扎实推进“十项民生工程”及20件民生大事。抓好高校毕业生、农民工等重点人群就业工作，确保新增城镇就业6万人以上，城镇登记失业率控制在4.4%以下。全面完成“全民参保登记计划”工作，加快完善覆盖城乡居民的社会保障体系，健全“五险”市级统筹机制，提高社会保障统筹质量。提高城乡低保救助标准，健全完善大病救助保障机制，全力保障困难群众基本生活。

　　切实强化公共服务。 大力支持西华师范大学创建博士学位授予单位、西南石油大学（南充）创办独立应用型工科大学、川北医学院建设高水平医科大学，加快筹建四川省医药卫生职业学院、四川文化旅游职业学院和四川建华职业学院（民办）；继续加强义务教育、学前教育和特殊教育，持续提升高中教育、职业教育和民办教育质量。加快推进基层医疗卫生信息系统和县级平台建设，全面完成与市级卫生信息平台对接，实现市、县、乡、村卫生资源共享。巩固提升国家公共文化服务体系示范区创建成果，精心筹办第二届中国（南充）国际木偶艺术周、四川省第三届残疾人文化艺术节、四川省农民艺术节等重大文化活动。

　　切实深化社会治理。 健全稳定风险评估、矛盾纠纷化解、突发公共事件应急处置等机制，实现政府治理和社会自我调节、居民自治良性互动。持续推进“法治南充”建设，切实抓好“七五”普法，深化“法律七进”。全面推动涉法涉诉问题与信访分离，引导群众依法逐级反映诉求，严格执行处理、复查、复核信访三级终结制度。层层压实安全生产责任，加强交通运输、建筑施工、工矿企业等领域安全监管，坚决遏制重特大事故发生。加强食品药品安全监管，保障群众“舌尖上的安全”。开展“平安南充”专项整治，加强防灾减灾救灾工作，全力维护社会和谐稳定。

　　（八）坚持为民施政、依法行政，切实加强政府自身建设

　　加快建设法治政府。 严格依照法定权限和程序行使权力、履行职责，提高依法行政、依法办事能力和水平。加大法治教育培训力度，增强公务人员法治意识。自觉接受市人大及其常委会的法律监督、工作监督和市政协的民主监督，主动接受司法监督、群众监督和舆论监督。推进依法科学民主决策，着力提高决策质量。实行重大行政执法决定法制审核等制度，严格行政执法过错责任追究，切实推进依法公正文明执法。

　　加快建设诚信政府。坚持办实事、说实话、求实效，严格履行向社会作出的承诺，切实做到言必行、行必果。健全信用体系，加强政务诚信、商务诚信、社会诚信建设。强化政府系统诚信教育和管理，加强社会诚信宣传，增强全民信用意识。建立诚信典型“红名单”、严重失信主体“黑名单”制度，让守信者受益、失信者受限，推动形成褒扬诚信、惩戒失信的良好氛围。

　　加快建设开放政府。按照“公开为常态、不公开为例外”的原则，拓宽政务信息公开渠道，深入推进决策、执行、管理、服务和结果“五公开”。加快建设政府网站群、网络问政平台，优化完善网上政务服务功能。有序扩大市政府全体会、常务会和专题会开放度，完善重大行政决策公众咨询制度，深入开展政府开放日活动，及时回应社会关切，促进施政与民意良性互动常态化。

　　加快建设效能政府。坚持“一事一主体、一主体一责任人”，实行部门责任公开承诺制，落实重点项目、重点工作跟踪督办和责任追究制。完善政府绩效管理体系，持续推行社会满意度测评。坚持“放管服”三管齐下，健全完善“三张清单、一张网”，着力提升行政服务效能。深入开展“庸懒散浮拖”专项整治，健全正向激励机制，让广大干部想干事、敢干事、干成事。构建“亲”“清”政商关系，营造优质高效政务环境。

　　加快建设廉洁政府。坚决贯彻从严治党、从严治政各项规定，认真落实党风廉政建设责任制，切实加强政府系统思想政治建设和反腐倡廉建设。严格执行中央“八项规定”和省委省政府“十项规定”，持续深化正风肃纪，坚决防止“四风”反弹。强化制度建设，扎紧制度“围栏”，堵住腐败“暗道”。加强行政监察和审计监督，做到有案必查、有腐必反、有贪必肃，巩固良好政治生态，树立良好政务形象。

　　各位代表！航程已开启，扬帆破浪正当时；任重而道远，策马扬鞭勇当先。让我们更加紧密地团结在以习近平同志为核心的党中央周围，在省委省政府和市委的坚强领导下，紧紧依靠和团结全市人民，以勇立潮头、勇攀高峰的激情，以义无反顾、一往无前的气魄，以踏石留印、抓铁有痕的干劲，冲锋在前、跨越在先，昼夜兼程、不胜不休，为开创南充新未来、建设成渝第二城、决战全胜同步全面小康而不懈奋斗！
